# Negative Colonoscopy After Positive Multitarget Stool DNA: Subsequent Aerodigestive Cancer Diagnosis and Recommendations in the Community Setting

**DOI:** 10.14309/ctg.0000000000001081

**Published:** 2026-08-17

**Authors:** Kaden R. Narayani, Raj I. Narayani, Kelli N. Burger, Douglas W. Mahoney, John B. Kisiel, Derek W. Ebner

**Affiliations:** 1 Gastrointestinal Associates, PC, Knoxville, Tennessee, USA;; 2 Division of Biomedical Statistics and Informatics, Mayo Clinic, Rochester, Minnesota, USA;; 3 Division of Gastroenterology and Hepatology, Mayo Clinic, Rochester, Minnesota, USA.

**Keywords:** early detection of cancer, colorectal neoplasms, gastrointestinal neoplasms, false-positive reactions, guideline adherence

## Abstract

**INTRODUCTION:**

Multitarget stool DNA (mt-sDNA) is a sensitive stool-based test for colorectal cancer screening. Limited referral-center data suggest low rates of incident aerodigestive cancers after a positive mt-sDNA test and negative colonoscopy; community-based outcomes are unknown.

**METHODS:**

Retrospective cohort study of adults referred for colonoscopy after positive mt-sDNA in a large community practice (2017–2021). Among patients with negative colonoscopy, we manually recorded postcolonoscopy recommendations, findings if additional colonoscopies had been performed, aerodigestive cancer diagnosis, and last clinical follow-up. Observed cancer incidence was compared with Surveillance, Epidemiology, and End Results data.

**RESULTS:**

Of 1,176 patients completing colonoscopy after positive mt-sDNA, 328 (28%) had negative findings. The median cohort age was 67 (interquartile range 59–72) years. Colonoscopy was performed at a median of 53 days after mt-sDNA sample collection. Postcolonoscopy recommendations aligned with Multi-Society Task Force (MSTF) guidelines in 59% of patients. Thirty-two patients underwent repeat colonoscopy with a median of 3.5 (1.9–4.4) years later; 2 (6.3%) had advanced serrated lesions, and no colorectal cancer was detected. The median clinical follow-up was 4.4 (3.4–5.6) years. Seven patients developed aerodigestive cancers (2 pancreatic, 5 lung), consistent with expected age- and sex-adjusted Surveillance, Epidemiology, and End Results incidence (n = 7.62) (*P* = 0.823).

**DISCUSSION:**

In this community cohort, when a positive mt-sDNA test was followed by negative colonoscopy, rates of subsequent advanced colorectal neoplasia and aerodigestive cancer were low. Most patients received recommendations for subsequent colonoscopy that were consistent with MSTF guidelines. Findings further support the current MSTF recommendation of no additional evaluation for extracolonic malignancy in asymptomatic patients after high-quality follow-up colonoscopy.

## Graphical abstract

**Figure:**
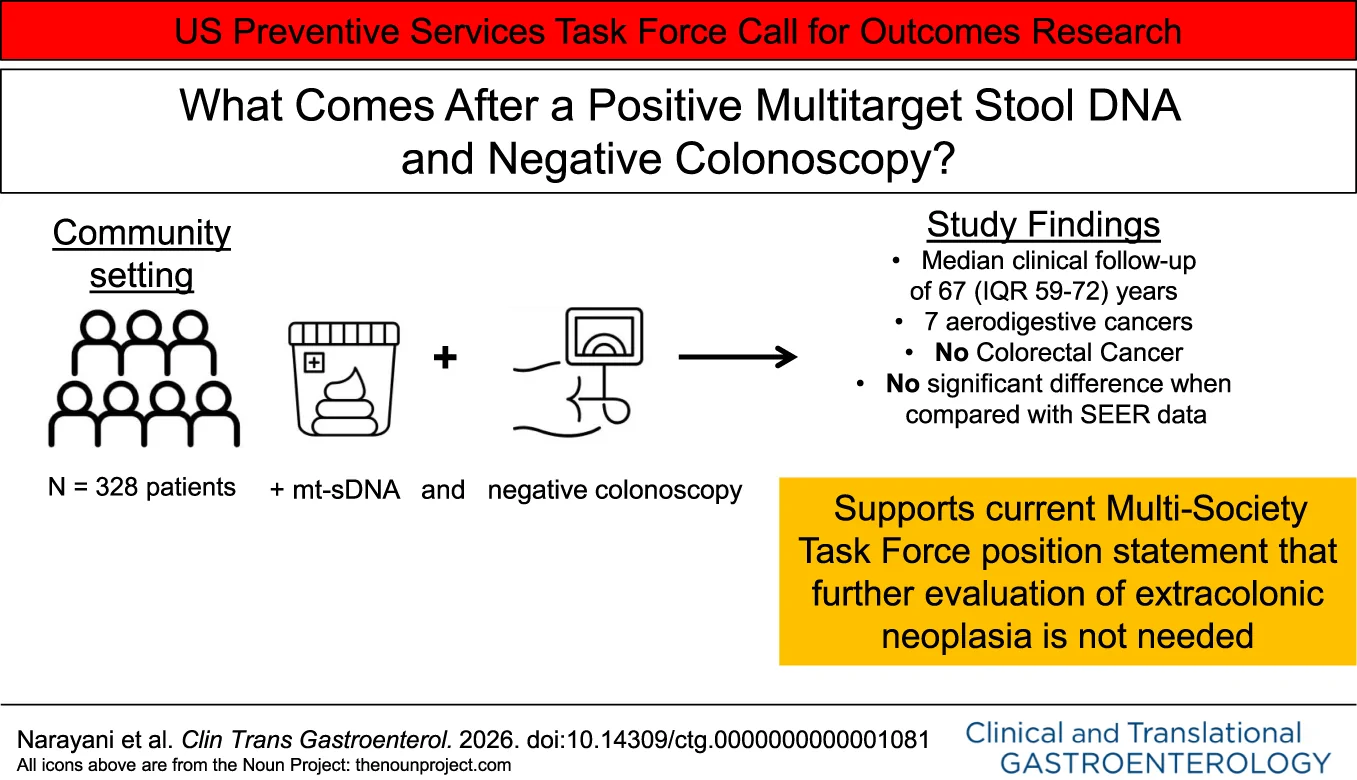

## INTRODUCTION

Colorectal cancer (CRC) is the second-leading cause of cancer-associated death in the United States (1). Screening colonoscopy reduces CRC incidence and mortality, but uptake can be limited by procedural concerns such as the need for bowel preparation, time off from work, concern for procedure-associated pain, and cost (2). Multitarget stool DNA testing (mt-sDNA; Cologuard, Exact Sciences, Madison, WI) is an US FDA-approved noninvasive CRC screening option for average-risk adults and endorsed by the US Preventive Services Task Force and US Multi-Society Task Force (MSTF) (3,4). The assay combines exfoliated molecular markers of neoplasia with fecal immunochemical testing, offering higher sensitivity than fecal immunochemical test alone (5). The advent and availability of mt-sDNA has fostered an increase in CRC screening uptake in the US population (6,7).

Noninvasive CRC screening is a two-step process. When the noninvasive screening test is positive, a follow-up colonoscopy is required to complete the screening paradigm, and subsequent care is dependent on the colonoscopy findings. In practice, when follow-up colonoscopy is negative (i.e., discordant test result), there may be uncertainty whether the result reflects a false-positive assay result or false-negative colonoscopy. A third possibility is a positive test result triggered through the detection of an occult extracolonic malignancy (8). Available data are limited to measure the rate of these events and the magnitude of these concerns for patients and providers (9–12). It is not surprising that with the novel assessment of tumor markers by mt-sDNA in conjunction with historically limited investigations of discordant results, the US Preventive Services Task Force has identified this as a research gap, specifically citing a need for “studies that report outcomes of patients who receive abnormal mt-sDNA results but subsequently negative colonoscopy results” (8).

Our primary objective was to identify patients with a positive mt-sDNA test and negative follow-up colonoscopy (no advanced or nonadvanced colorectal neoplasia) and discern the incidence of advanced colorectal neoplasia (advanced precancerous lesions or CRC), aerodigestive cancers, and death. Second, we measured the rate of endoscopist adherence to MSTF guideline recommendations for repeat screening after a positive mt-sDNA test but negative colonoscopy result. By comparing cancer incidence rates to Surveillance, Epidemiology, and End Results (SEER) population data, we hoped to provide empirical evidence to guide clinicians and patients in this real-world clinical situation.

## METHODS

A retrospective cohort study was performed in a large, single-specialty community gastroenterology practice serving the Knoxville metropolitan area and surrounding East Tennessee referral region. The practice includes 16 physicians, 8 advanced practice providers, 3 pathologists, 3 ambulatory surgery centers, and coverage of one 468-bed hospital. The catchment area represents approximately 34 counties, 13,500 square miles, and 2.6 million residents. Electronic health records (EHR) were reviewed for all consecutive positive mt-sDNA referrals to our gastroenterology practice from January 1, 2017, through December 31, 2021. All positive mt-sDNA referrals were seen through office consultation rather than open access colonoscopy. The study was approved by Covenant Health Institutional Review Board (IRB: 23-006998 INV Exempt). All positive mt-sDNA referrals were abstracted from gGastro EHR (Modernizing Medicine, Boca Raton, FL) by manual chart review. No formal sample-size or power calculation was performed because this retrospective cohort included all eligible patients from the practice during the study period. The cohort size was, therefore, determined by the available clinical population rather than by prospective enrollment. We searched and extracted clinical follow-up information from both gGastro EHR and East Tennessee Health Information Network (etHIN, Maryville, TN). Among referrals who then had a negative colonoscopy, we recorded (i) colonoscopy findings (neoplasia, cecal intubation, and bowel preparation quality), (ii) CRC risk factor status, (iii) postcolonoscopy rescreening/surveillance recommendation made, (iv) rescreening/surveillance colonoscopy findings (if performed), (v) date and stage of colorectal or aerodigestive cancer diagnosis (established through review of available clinical notes and patient problem list), and (vi) last clinical evaluation and vitality status of each subject. Regarding CRC risk factor status, high-risk referred to a patient with inflammatory bowel disease, personal history of advanced colorectal neoplasia or CRC, a known genetic syndrome predisposing to CRC, first-degree relative with CRC diagnosed aged 60 years or younger, or the patient having signs or symptoms of bleeding (+FOBT [<6 months], overt rectal bleeding [<3 months], or iron deficiency anemia [<3 months]). Aerodigestive cancer locations were consistent with those categorized by SEER, as noted below. We entered data into a secure REDCap database (Vanderbilt University, Nashville, TN) hosted by Mayo Clinic. Observed incidence rates were compared with expected incidence based on SEER data.

Clinical and demographic information was summarized as a median and intraquartile range for continuous variables and as a percentage of subgroup totals for categorical variables. Given the fixed sample and small number of outcome events, the results are reported descriptively with confidence intervals and no formal power calculations were conducted *a priori*. The primary end point of analysis is the incidence rate of aerodigestive cancers after a positive mt-sDNA stool test but negative colonoscopy. Time zero for this analysis was the date of mt-sDNA testing and censoring occurred at the date of last clinical follow-up or death. We summarized the incidence of aerodigestive cancers as a function of time using the methods of Kaplan and Meier (13). To compare the observed incidence of aerodigestive cancer with that expected in the general population, SEER incidence data for 18 cancers (lip, tongue, salivary gland, floor of mouth, gum, nasopharynx, tonsil, oropharynx, hypopharynx, esophagus, stomach, small intestine, colon/rectum, pancreas, gall bladder, bile duct, hepatocellular, and cholangiocarcinoma of the liver) were compiled for the years spanning 2012 to 2021. The expected rate of aerodigestive cancers to that of an age- and sex-matched population-based cohort was estimated using methods described by Ederer et al (14) with corresponding 95% confidence intervals.

## RESULTS

Among 1,294 patients referred during the target period for colonoscopy after a positive mt-sDNA test, 1,176 (91%) completed the procedure. A negative colonoscopy was documented in 328 patients (28%) and was performed a median of 53 days after referral (interquartile range [IQR], 35–84 days). The median age of patients with a negative colonoscopy was 67 years (IQR, 59–72), 35% were male (n = 116), and 98% were White (n = 323) (Table 1). There were 31 patients (9%) who used mt-sDNA but were classified as high risk for CRC: 1 patient had iron deficiency anemia within 90 days before mt-sDNA testing; 27 patients had a first degree family member with CRC aged 60 years or younger; 1 had inflammatory bowel disease; and 2 patients had a personal history of advanced colorectal neoplasia (before mt-sDNA). The majority of patients, 174 (53%), had a history of previous colonoscopy (see Supplementary Table 1, Supplementary Digital Content, https://links.lww.com/CTG/B583). The median clinical follow-up for the cohort was 4.4 (3.4–5.6) years.

**Table 1.** Patient characteristics among those with positive mt-sDNA test and negative follow-up colonoscopy

Patient characteristic	Positive multitarget stool DNA and negative colonoscopy N = 328
Median age, yr, IQR	67 (59–72)
Men, n (%)	116 (35%)
Race	
White	323 (98%)
Black	3 (1%)
Asian	2 (1%)
Tobacco	
Current	50 (15%)
Former	121 (37%)
Never	157 (48%)

After a negative colonoscopy, 59% of patients received a recommendation for repeat screening in accordance with the MSTF guidelines (Table 2). Recommendations that failed to follow MSTF guidelines were most commonly observed among a small subset of the practice's physicians who universally advised more conservative recommendations after negative colonoscopy. Another physician consistently failed to advise any procedure follow-up interval in patients aged older than 65 years. Among the 31 patients who used mt-sDNA even though their personal or family history rendered them high risk before testing, 81% (25/31) were recommended to have a colonoscopy within 5 years of the negative follow-up colonoscopy, 13% (4/31) were recommended to defer additional screening based on age, 1 patient was directed to barium enema, and 1 patient did not have a recommendation listed. Across the entire cohort, 32 patients underwent a subsequent colonoscopy at a median interval of 3.5 years (IQR, 1.9–4.4 years) after their original mt-sDNA follow-up colonoscopy; of these, 2 patients (6.3%) were found to have advanced serrated lesions (both patients were at average risk before mt-sDNA use) and none had CRC.

**Table 2.** Recommendations after negative follow-up colonoscopy

Recommendation	Frequency, n (%) ​
Total	328 (100)
Colonoscopy in 10 yr	154 (46.9)
No further screening based on age	29 (8.8)
Colonoscopy in 5 yr based on family history	5 (1.5)
Colonoscopy in < 3 mo due to inadequate bowel prep	4 (1.2)
Repeat colonoscopy in	
7–10 yr	1 (0.3)
5 yr	60 (18.3)
3 yr	14 (4.3)
2 yr	3 (0.09)
1 yr	7 (2.1)
Less than 1 yr	3 (0.09)
Barium enema or computerized tomography colonography	5 (1.5)
Not specified	43 (13.1)

Shaded recommendations signify those that are consistent with Multi-Society Task Force recommendations.

During the study period, 7 patients were diagnosed with subsequent aerodigestive cancer (5 average-risk patients and 2 high-risk patients). There were 2 cases of pancreatic adenocarcinoma (stages III and IV) and 5 cases of lung cancer (adenocarcinoma [stage IB], large cell neuroendocrine carcinoma [stage IIIA], non-small-cell carcinoma [stage IIIA], small-cell carcinoma [stage IV], and neuroendocrine tumor [stage IA]). Time to aerodigestive cancer diagnosis after the negative mt-sDNA test was a median of 2.3 (IQR, 0.4–4.3) years. Based on SEER incidence rate data, 7.62 aerodigestive cancers were expected from a similar age- and sex-matched population, with no statistically significant difference between observed and expected incidence overall (*P* = 0.8230) (Figure 1). The overall estimated aerodigestive event rate was 5.0 per 1,000 person-years (95% CI: 2.0–10.3 per 1,000 person-years). When stratified by average- vs. high-risk, the estimated aerodigestive event rates were 3.9 per 1,000 person-years (95% CI: 1.3–9.2 per 1,000 person-years) and 16.2 per 1,000 person-years (95% CI: 2.0–58.6 per 1,000 person-years), respectively. These were not found to be statistically significant (*P* = 0.2190), primarily due to the limited number of high-risk patients within the cohort.

**Figure 1.**
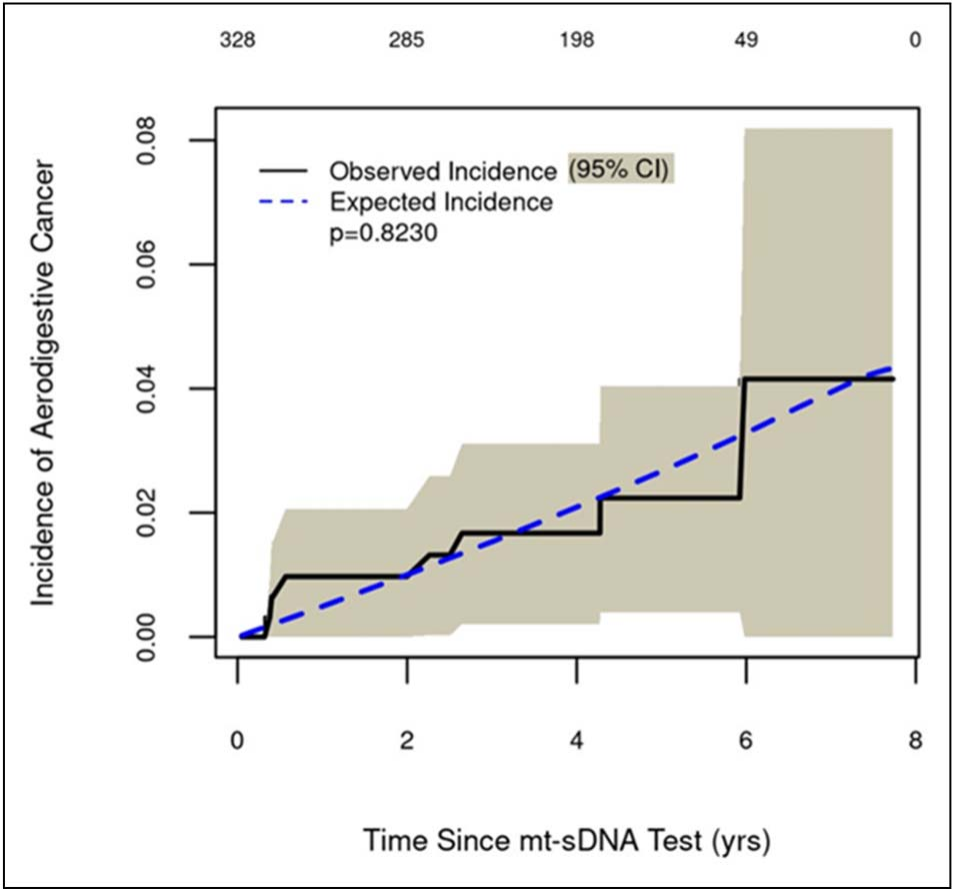
Observed aerodigestive cancers in the community compared with the expected incidence per Surveillance, Epidemiology, and End Results data.

## DISCUSSION

In this large, community-based cohort study of patients with a positive mt-sDNA test followed by a negative colonoscopy, the subsequent incidence of advanced colorectal neoplasia was low, and no cases of CRC were detected during follow-up. Furthermore, the observed rate of extracolonic aerodigestive cancers was statistically indistinguishable from population-based estimates derived from the SEER program.

These findings reinforce previous referral-center reports. One of the earliest referral-center reports evaluated 3 preapproval assay versions in the development of mt-sDNA and explored whether false-positive tests were linked to greater mortality and subsequent cancer incidence (9). In their experience, Cotter et al found 5-year survival to be similar among false-positive and true-negative mt-sDNA users; however, the cancer incidence was less than expected based on SEER data. A later study by Berger et al found aerodigestive cancer incidence to be no different among individuals with a negative colonoscopy independent of mt-sDNA result, and when compared with SEER data, the incidence of aerodigestive cancer was less among both groups (negative colonoscopy and either positive or negative mt-sDNA) (10). The most recent study was a prospective evaluation of 12 patients with positive mt-sDNA and negative colonoscopy who later underwent repeat mt-sDNA testing with colonoscopy and upper endoscopy, and no malignancy was identified (11). A concern with academic referral-center studies is whether the patient population is generalizable to the community level. Our study findings provide additional support for the guideline recommendation that no additional extracolonic evaluation is warranted for asymptomatic individuals who have positive mt-sDNA, followed by a high-quality colonoscopy (cecal intubation, adequate bowel preparation, appropriate withdrawal time, and a proceduralist with adequate neoplasia detection rates) (12). Other studies and societal guidelines have historically concluded the same for patients with a negative colonoscopy after a positive fecal immunochemical test (15–17).

Among those with positive mt-sDNA, the proportion of negative colonoscopy results in our study (28%) is consistent with previous real-world reports (9–11), underscoring that this is a reproducible phenomenon in community practice. On follow-up colonoscopy, only 6.3% of patients undergoing repeat evaluation had advanced serrated lesions, and none had CRC at a median interval of 3.5 years (IQR, 1.9–4.4 years) after their original mt-sDNA follow-up colonoscopy. Realizing only 32 patients of the cohort underwent the subsequent colonoscopy, we have limited power to delineate subsequent colorectal neoplasia risk for a false-positive mt-sDNA. Furthermore, it would be challenging to delineate future colorectal neoplasia rates after a false-positive mt-sDNA in a real-world retrospective cohort study as patients would not be anticipated to undergo repeat CRC screening for 10 years. Interestingly, our rate of advanced neoplasia was lower than that seen in the study by Cooper et al in which the advanced neoplasia rate was 17% (2/12), 11–29 months after the initial positive multitarget stool DNA follow-up colonoscopy (11). However, our findings align closely with the findings by Berger et al, (10) who reported no difference in aerodigestive cancer incidence between patients with positive versus negative multitarget stool DNA when colonoscopy was negative. In our analysis, the 7 aerodigestive cancers detected (predominantly lung) over more than 4 years of follow-up were consistent with the 7.62 cases predicted by SEER, suggesting no excess cancer signal beyond background risk. Thus, a negative colonoscopy after positive mt-sDNA test results reflects either a false-positive molecular detection or a missed biologically indolent lesion. Discernment of the 2 might require serial invasive colonoscopy in both mt-sDNA positive and negative patients for which the likely benefit is small relative to obvious risks and is well beyond the scope of this work.

We observed that only 59% of patients received postcolonoscopy screening recommendations consistent with MSTF guidelines (8,12), with some receiving accelerated follow-up without clear indication and others lacking documented guidance. Considering these patterns of aberrancy, even among gastroenterologists with vast clinical experience, the need for continuous education regarding appropriate screening or surveillance intervals cannot be overstated. Although patient-specific risk factors (e.g., family history, suboptimal bowel preparation) can justify shorter intervals, these findings highlight an opportunity for provider education to improve confidence and consistency in MSTF-directed rescreening colonoscopy recommendations when a positive mt-sDNA test is followed by a negative colonoscopy. Realizing the number of noninvasive cancer detection tests is increasing, research is needed to better understand what factors may contribute to recommending aggressive follow-up/evaluation after a false-negative test. Our study was not designed to further explore justifications for postcolonoscopy recommendations after positive mt-sDNA test and negative colonoscopy. However, this was the primary aim of Hakim et al who found only 51% of proceduralists recommended 10-year follow-up after a positive mt-sDNA test and negative colonoscopy (18). Interestingly, their study found that increased age and multiple comorbidities were associated with sooner follow-up colonoscopy recommendations.

Our study findings add to the limited but growing body of evidence supporting the MSTF recommendations that no further extracolonic workup is needed in asymptomatic patients after adequate negative colonoscopy, regardless of mt-sDNA result. From a practical standpoint, focusing on adherence to guideline-based rescreening intervals rather than pursuing low-yield additional imaging may reduce patient anxiety, prevent unnecessary testing, and optimize healthcare-associated resource utilization.

Our work provides the first evaluation of aerodigestive cancer rates among patients with positive mt-sDNA test and negative colonoscopy, exclusively in community practice. Our single-center experience may limit generalizability but does address the call for outcomes research among those with positive mt-sDNA test results and negative follow-up colonoscopy (8). Our cohort consisted predominantly of White women (Table 1). Cancer incidence is known to vary by sex and ethnicity, and although overall cancer incidence is highest among White individuals, variability tends to be cancer site-specific. Several aerodigestive cancers, including liver and CRC, have higher incidence rates among Black individuals and American Indian/Alaska Native populations. Similarly, lung cancer incidence among men is higher in the same ethnic groups. Consequently, our findings may not be generalizable to more diverse patient populations and should warrant further study. An additional limitation of our work is the retrospective design. As such, capture of an aerodigestive cancer diagnosis was only possible when described in the available medical record. Patients who developed cancer and sought care at an outside academic institution may have been missed, thus risking an underestimate of the cancer incidence. However, the diagnosis would likely have been captured within the primary care notation and with the practice patterns in our community, referring providers often communicate with our practice when a patient develops cancer, particularly if digestive. Furthermore, our manual review showed that 80% of the cohort had follow-up data beyond January 1, 2024, and therefore remained in the local/regional area. Overall, we suspect our rate of missed aerodigestive cancers is low, especially as the incidence rates in our study are similar to what was observed for prospective evaluations (9,10). Future investigators should consider utilization of patient survey in an effort to increase capture of study and points and protect against some of the limitations of retrospective EHR data; this, however, was beyond our study design and study resources. Our sample size limited greater precision but provides the largest community-based series to date. It is also important to note that the anticipated frequency of a positive mt-sDNA test and negative colonoscopy is also low (13%) in light of the high-test performance of mt-sDNA (5). Although a strength of our analysis is its comparison with an external control (SEER), adjustment was limited to age and sex. Other important confounders, such as smoking status, were not included. This is particularly important if aerodigestive cancer rates exceed those expected based on SEER data, as smoking could substantially influence these findings. Future studies should adjust for additional confounders, including smoking status, and compare aerodigestive cancer incidence with both SEER data and negative screening controls from the same institution to strengthen confidence in observed differences.

Overall, we conclude that in this sample of patients with a positive mt-sDNA test followed by negative colonoscopy, observed rates of subsequent advanced colorectal neoplasia, CRC, and extracolonic aerodigestive cancer were low and/or comparable with those expected in the general population. These findings extend previous referral-center experiences into real-world community practice, supporting guidelines that argue against additional extracolonic evaluation. Improving adherence to MSTF recommendations for rescreening intervals represents a tangible opportunity to enhance care consistency in this setting. Future research would ideally include a large series of patients with at least 3 years of follow-up where the rate of aerodigestive cancers is compared with patients having other CRC screening modalities.

## CONFLICTS OF INTEREST

**Guarantor of the article:** Derek W. Ebner, MD.

**Specific author contributions:** K.R.N.: data curation, investigation, writing—original draft, review and editing, lead. R.I N.: data curation, investigation, writing—review and editing, supporting. K.N.B.: data curation, formal analysis, project administration, writing—review and editing, supporting. D.W.M.: formal analysis, methodology, validation. writing—review and editing, supporting. J.B.K.: conceptualization, funding acquisition, methodology, supervision, writing—review and editing, supporting. D.W.E.: conceptualization, funding acquisition, data curation, investigation, validation, writing—review and editing, supporting.

**Financial support:** This work was supported by a grant from the division of Gastroenterology and Hepatology at Mayo Clinic, Rochester, MN awarded to D.W.E. and a grant from the National Cancer Institute (CA214679) awarded to J.B.K.

**Potential competing interests:** D.W.M. and J.B.K. are listed as inventors of Mayo Clinic intellectual property licensed to Exact Sciences and have rights to receive royalties, paid to Mayo Clinic. D.W.E. has a professional service agreement with Exact Sciences serving as an independent contractor to provide guidance on study design and analysis, paid to Mayo Clinic. The remaining authors do not have any competing interests to report.

**Study Highlights**

**WHAT IS KNOWN**

✓ Multitarget stool DNA (mt-sDNA) screens for colorectal cancer through detection of hemoglobin and exfoliated tumor markers and, when positive, requires follow-up colonoscopy.
✓ USPSTF has called for research on the outcomes among asymptomatic patients with a positive mt-sDNA test and negative colonoscopy due to limited data on extracolonic cancer incidence for these patients.
✓ Observational data on favorable outcomes for individuals with a positive mt-sDNA test and negative colonoscopy are limited to referral settings.

**WHAT IS NEW HERE**

✓ Incidence rates of aerodigestive cancer among patients with a positive mt-sDNA test and negative colonoscopy are low in the community setting.
✓ The observed cancer rates are consistent with expected rates when compared with Surveillance, Epidemiology, and End Results data.
✓ There is an opportunity for provider education to improve confidence and consistency in Multi-Society Task Force-directed rescreening colonoscopy recommendations.

## Supplementary Material

## ABBREVIATIONS:

CRC: colorectal cancer
EHR: electronic health records
IQR: interquartile range
MSTF: multi-society task force
mt-sDNA: multitarget stool DNA
SEER: surveillance, epidemiology, and end results
