# Supplementary material for: Negative Colonoscopy After Positive Multitarget Stool DNA: Subsequent Aerodigestive Cancer Diagnosis and Recommendations in the Community Setting
Source: Clin Transl Gastroenterol. 2026 Aug 17;17(10):e01081. doi: 10.14309/ctg.0000000000001081 (PMC13694089; doi:10.14309/ctg.0000000000001081)
Supplement: Supplementary file 1 [file ct9-17-e01081-s001.docx]

**Supplementary Table 1**

| Colonoscopy finding | Patients with prior colonoscopy  N= 174 |
| --- | --- |
| Report not available for review | 51 (29%) |
| Negative for colorectal neoplasia | 99 (30%) |
| Positive for colorectal neoplasia* | 24 (14%) |

*Among those with colorectal neoplasia at prior colonoscopy: 2 patients had advanced precancerous lesion (advanced adenomas measuring >= 1 cm in the greatest dimension or with high-grade dysplasia or villous elements), 1 had 1-2 sessile serrated polyps 5 - < 10 mm, 5 had >=3 adenomas < 10 mm, non-advanced, 15 had 1-2 adenomas < =5 mm, non-advanced and 1 had 1-2 sessile serrated polyp < =5 mm, non-advanced.

**Supplemental Table 2**

Frequency of colonoscopy preparation quality and cecal intubation

Positive mt-sDNA and follow-up colonoscopy, N= 328

| Preparation Quality | Cecal intubation | |  |
| --- | --- | --- | --- |
|  | No | Y | Unknown |
| Unknown | - | 2 | 2 |
| Inadequate | 2 | 6 |  |
| Fair |  | 6 |  |
| Adequate | 7 | 302 | 1 |

Supplemental Table 3

Gastroenterologists' years from fellowship training during the study period by year

| Year | 2017 | 2018 | 2019 | 2020 | 2021 |
| --- | --- | --- | --- | --- | --- |
| Number of gastroenterologists | 13 | 14 | 13 | 16 | 15 |
| Median number of years from training (range) | 22 (3-31) | 20 (0-32) | 18 (1-33) | 19 (0-34) | 20 (1-34) |
